# Impact of Sex on Lung Function in Adult Langerhans Cell Histiocytosis

**DOI:** 10.3390/life15081258

**Published:** 2025-08-07

**Authors:** Antonio Fabozzi, Gianluca Paciucci, Giulia de Rose, Roberto Romiti, Giovanna Palumbo, Gregorino Paone, Matteo Bonini, Paolo Palange

**Affiliations:** 1 Pulmonology Unit, Department of Public Health and Infectious Diseases, Policlinico Umberto I, “Sapienza” University of Rome, 00185 Rome, Italy; paciucci.gianluca@gmail.com (G.P.); giulia.derose@yahoo.it (G.d.R.); roberto.romitimp@gmail.com (R.R.); gregorino.paone@uniroma1.it (G.P.); matteo.bonini@uniroma1.it (M.B.); paolo.palange@uniroma1.it (P.P.); 2 Division of Hematology, Department of Translational and Precision Medicine, Policlinico Umberto I, “Sapienza” University of Rome, 00185 Rome, Italy; palumbo@bce.uniroma1.it

**Keywords:** interstitial lung diseases, diffuse parenchymal lung diseases, lung function tests, respiratory function test, computed tomography, Langerhans Cell Histiocytosis, Pulmonary Langerhans Cell Histiocytosis, sex differences, female characteristics

## Abstract

Background: Langerhans Cell Histiocytosis (LCH) is a rare histiocytic hematological disorder that frequently involves the lungs. Due to a lack of data about sex-related differences in LCH, the aim of this study is to evaluate sex-related differences in pulmonary function in a cohort of patients with LCH. Methods: We retrospectively analyzed data from 79 adult patients diagnosed with LCH. Demographic, clinical, and spirometric data were collected and compared by sex. Continuous variables were analyzed using the Mann–Whitney test and categorical variables were analyzed with the Chi-square test. Results: Out of 79 patients, 47 (59.5%) were females and 32 (40.5%) were males. Women showed significantly lower diffusing capacity of the lungs for carbon monoxide (DLCO%) and lower diffusing capacity of the lungs for carbon monoxide per unit of alveolar volume (DLCO/VA%) compared to men. Females showed a trend toward lower small airway indices, including maximal expiratory flow at 25 (MEF_25_%) and forced expiratory flow at 25–75% (FEF_25–75_%), though this was not statistically significant, while the residual volume-to-total lung capacity (RV/TLC) ratio was significantly higher in women. Among the functional parameters, DLCO% showed the highest accuracy (AUC 0.70) in the identification of lung involvement after multivariate regression analysis. Conclusions: Our findings suggest that the combination of lower gas exchange efficiency and increased peripheral air trapping secondary to small airway involvement in female patients may reflect the presence of a distinct functional LCH phenotype in women characterized by early small airway involvement and altered ventilation–perfusion dynamics, which may influence the clinical management of these patients. Furthermore, the moderate predictive value of DLCO% for lung involvement at baseline in LCH women suggests that DLCO may contribute to the detection of LCH women with lung involvement, although it should not be considered a definitive diagnostic test without a prospective and independent external validation.

## 1. Introduction

Langerhans Cell Histiocytosis (LCH) is a rare hematological neoplasm of a histiocytic nature with multisystemic involvement [1]. The most frequently affected sites include the bones, lungs, lymph nodes, spleen, and liver [2]. The incidence of LCH is approximately 1–1.5 cases per million per year, according to the latest data [3]. When LCH affects only a single organ, it is classified as Single-System LCH (SS-LCH), with lung involvement defined as Pulmonary Langerhans Cell Histiocytosis (PLCH), accounting for approximately 32% of all LCH cases [4,5]. The pathogenesis of LCH is not yet fully understood but appears to involve the clonal proliferation of Langerhans cells (LCs) and CD1a+ myeloid precursor cells, often driven by the mutation of the MAPK pathway [6,7,8]. In PLCH, the infiltration of peribronchiolar granulomas caused the destruction of the small airways, leading to air trapping and ventilation–perfusion impairment [9]. Notably, tobacco and marijuana smoking are well-established risk factors for PLCH’s development [10,11]. From a functional perspective, PLCH is associated with impairment in lung function, although approximately 12–20% of PLCH patients exhibit normal Pulmonary Function Tests (PFTs) at diagnosis [12,13,14]. Case series report a prevalence of obstructive ventilatory deficit in PLCH of around 16–40%, with a restrictive ventilatory deficit between 2.5% and 25.7% and mixed ventilatory deficit of around 2.7–33% [12,13,14,15,16]. PLCH course is heterogeneous: up to 38% of patients develop deterioration of lung function at 24 months (defined as a reduction of ≥15% in FEV_1_, FVC, or DLCO) [16]. However, the most common finding at PFTs remains the reduced diffusing capacity of the lungs for carbon monoxide (DLCO), present in approximately 70–90% of cases [12,13,14,15]. Furthermore, a Chinese case series reported isolated reduced DLCO as the only pathological finding in PFTs in 44% of PLCH patients [16].

The impact of sex on lung function, clinical outcomes, and prognosis is well known in other interstitial lung diseases (ILDs) [17]. In a Swedish case series, women with Idiopathic Pulmonary Fibrosis (IPF) had higher %FVC and %TLC than men. Furthermore, men with IPF have a poorer prognosis, as demonstrated by the higher Gender–Age–Physiology (GAP) score for men (1 point) than for women (0 points) [18]. A recent Italian retrospective study also showed that men had lower FVC% at presentation and reduced 5-year survival compared to women [19]. However, other studies did not show that the female sex was associated with better survival in IPF after adjusting for age and %FVC at presentation [20]. In sarcoidosis, conversely, women have greater extrapulmonary involvement (musculoskeletal, ocular, and cutaneous), a higher frequency of Pulmonary Arterial Hypertension (PAH), a poorer quality of life, and higher mortality than men [21,22,23,24,25]. Therefore, despite the known impact of sex on clinical features and prognosis in other ILDs, its role in LCH or PLCH remains poorly understood. For this reason, this study aims to evaluate the influence of sex on lung function in adult LCH at baseline. Specifically, our aim is to identify possible sex-based functional phenotypes in LCH that may provide new key concepts in pathophysiology and clinical decision-making.

## 2. Material and Methods

This study is a retrospective and observational one that was performed at the Interstitial Lung Diseases Center, Pulmonology Unit, Policlinico Umberto I, “Sapienza” University of Rome (Italy). The study retrospectively included all 79 adults with a histological diagnosis of LCH who were referred to our center for their first pulmonology evaluation from January 2020 to July 2024. The inclusion criteria included the following: age between 18 and 80 years old, histologically confirmed diagnosis of LCH and the execution of Pulmonary Functions Tests (PFTs), and the diffusing capacity of the lungs for carbon monoxide (DLCO) and Multiple-Breath Nitrogen Washout (N2-MBW) tests at the first visit. The exclusion criteria included the presence of chronic respiratory diseases (such as Chronic Obstructive Pulmonary Disease [COPD], asthma, and bronchiectasis), neuromuscular diseases potentially affecting the respiratory system, severe heart failure (NYHA ≥ III), and active cancer or hematologic disease other than LCH at the time of inclusion in the study. Demographic data (age, sex, smoking habit, and pack years), clinical data (symptoms), and functional data from the electronical records in the spirometer were collected and stratified by sex. Lung involvement in patients with a histological diagnosis of LCH was defined based on HRCT images reviewed by an experienced chest radiologist using the 2024 position statement from the UK Cystic Lung Disease Rare Disease Collaborative Network [26]. The patients’ characteristics are summarized in Table 1.

PFTs, DLCO, and N2-MBW were measured using a spirometer (Quark PFT, Cosmed, Pavona, Italy), according to the American Thoracic Society and European Respiratory Society guidelines [27,28,29] and Global Lung Initiative for technical standards and reference values [30]. The spirometer was calibrated for at least 3 cycles every day using a 3-liter syringe. The tolerance error was ≤75 mL. A gas analyzer for DLCO and M2-NBW was used before each test. All PFTs, DLCO, and N2-MBW tests were performed, analyzed, and validated by the same trained pulmonary physiologist to minimize inter-observer variability. Bias gas airflow equipment dispensed a valve-controlled delivery of 100% medical oxygen during the N2-MBW test. In order to start the test, the subjects breathed calmly through a mouthpiece while keeping their nose closed. After approximately five normal breaths of room air, the equipment automatically switched to oxygen output at the end of the expiration. By inhaling 100% medical oxygen, nitrogen was gradually removed from the lungs breath by breath. The wash-out phase was completed when the nitrogen level reduced to less than 1/40th of the initial level. The functional residual capacity (FRC) was determined by dividing the total volume of nitrogen exhaled by the difference in the concentration of nitrogen exhaled at the start and at the end of the wash-out phase.

For statistical analysis, continuous variables were analyzed using the Mann–Whitney test due to non-normal distribution; categorical variables were compared using the Chi-square test. Linear correlation between continuous variables was performed using Pearson’s correlation. Multivariate logistic regression analysis was conducted to identify independent predictive factors of lung involvement. A *p*-value < 0.05 was considered statistically significant. The statistical software we used was Jamovi version 2.6.44.0.

## 3. Results

**Figure 1:**
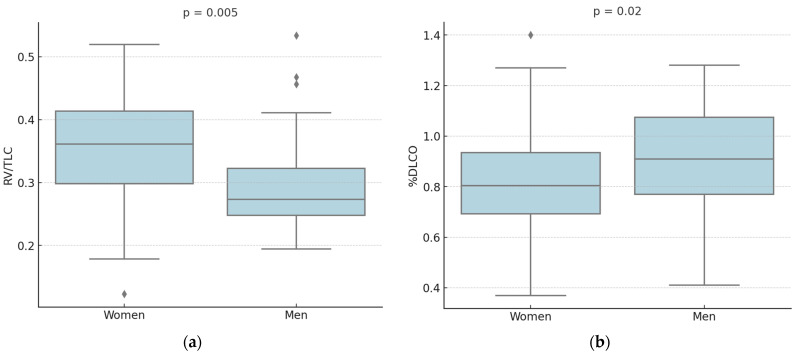
Pulmonary Function Tests’ differences between men and women. (**a**) Women showed a higher RV/TLC ratio (0.35 ± 0.09 vs. 0.3 ± 0.08, *p* = 0.005); (**b**) women presented lower %DLCO (81 ± 22% vs. 92 ± 24%, *p* = 0.02).

In total, 79 patients with a histological diagnosis of LCH (32 men and 47 women) were retrospectively recruited (Table 1). Men were younger than women (mean age: 38 ± 12 vs. 45 ± 12 years, *p* = 0.007) and reported a higher number of pack years (22 ± 21 vs. 12 ± 14, *p* = 0.02). The prevalence of lung involvement in the two cohorts was similar (72% vs. 62%). From a clinical perspective, more females reported exertional dyspnea compared to men (56.5 vs. 34.5%), without reaching statistical significancy (*p* = 0.3). From a functional perspective, men predictably exhibited higher absolute values in liters than women, due to their larger body size and muscle mass, as well as larger chest. At spirometry, women tended to report more small airway impairment with lower forced expiratory flow at 25–75% (FEF_25–75_%) and maximal expiratory flow at 25% (MEF_25_%), but the differences did not reach statistical significance compared to men (*p* = 0.075 for MEF_25_% and *p* = 0.08 for FEF_25–75_%). At N2-MBW, women showed a higher residual volume (RV)/ total lung capacity (TLC) ratio (0.35 ± 0.09 vs. 0.3 ± 0.08, *p* = 0.005), although neither %RV nor %TLC differed significantly between the two sexes (Figure 1). Finally, women reported lower %DLCO (81 ± 22% vs. 92 ± 24%, *p* = 0.02) and %DLCO/alveolar volume (VA) (84 ± 18 vs. 94 ± 18, *p* = 0.01) (Figure 1).

**Figure 2:**
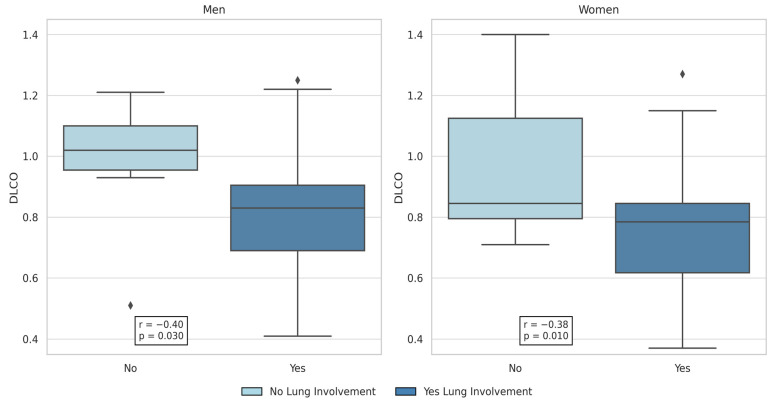
Univariate analysis showed inverse correlation between DLCO and lung involvement in LCH patients (both the cohorts: r = −0.4 and *p* = 0.03 for men; r = −0.38 and *p* = 0.01 for women).

In Pearson’s correlation, %DLCO was the only functional variable statistically significantly negatively associated with the presence of lung involvement in both sexes (r = −0.40 and *p* = 0.03 for men; r = −0.38 and *p* = 0.01 for women) (Figure 2).

**Figure 3:**
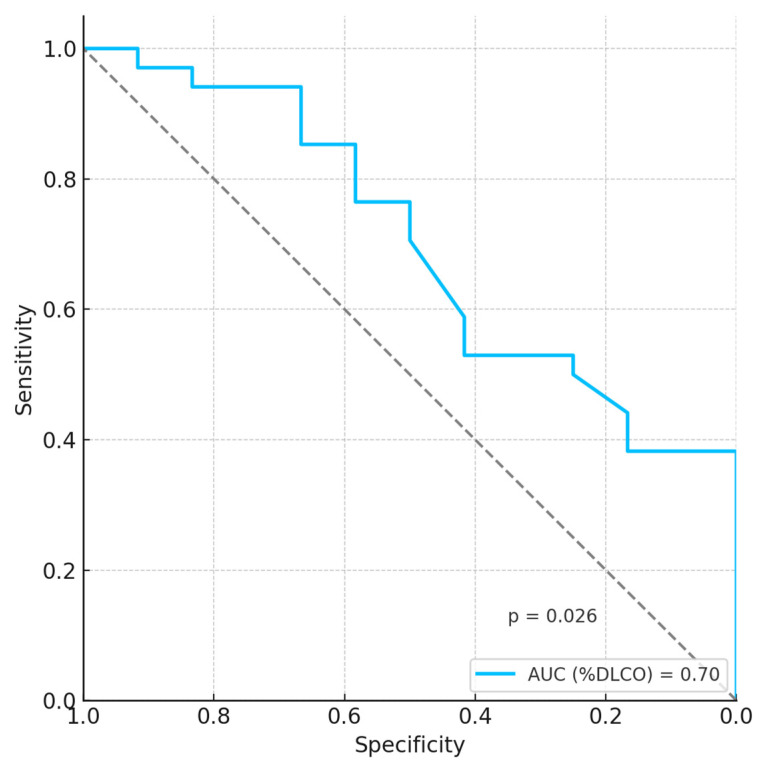
ROC curve showed that DLCO had moderate predictive power for lung involvement in LCH women (AUC: 0.70, *p* = 0.026).

The multivariate analysis, performed with adjustment for age, smoking habit, and pack years, showed that the only functional parameter predictive of pulmonary involvement was %DLCO in women only (Table 2 and Table 3). The ROC curve showed that %DLCO has a moderate ability to distinguish lung involvement in women with LCH (AUC: 0.70) (Figure 3).

## 4. Discussion

To date, this is the first study to provide interesting data on the effect of sex in a rare disease such as LCH, focusing on lung function and functional sex-related phenotypes. The first insight emerging from our study is that women diagnosed with LCH arrive at their first pulmonological evaluation at an older age compared to men (45 ± 12 vs. 38 ± 12 years). This finding aligns with previous data from the literature suggesting a delayed disease onset in females [31,32]. The prevalence of lung involvement in our LCH cohort was comparable between the two sexes, standing at 72% for women and 62% for men. These percentages are slightly higher than those reported in the literature, which indicate that 58% of LCH patients present with lung involvement [33].

### 4.1. Small Airway Involvement

From a functional perspective, women with LCH exhibited greater small airway dysfunction, as evidenced by the trend of lower ME_F25_% and FEF_25–75_% values in the women of our cohort. Small airway impairment, leading to premature collapse of the distal bronchioles during forced expiration, results in early air trapping [34]. The higher RV/TLC observed in LCH women further supports this pathophysiological mechanism, indicating a more severe small airway involvement. Histopathologically, these functional features may reflect peribronchiolar inflammation and concentric fibrotic remodeling of the respiratory bronchioles, driven by the uncontrolled clonal proliferation of Langerhans cells (LCs) within the bronchiolar walls and adjacent alveolar spaces [35].

Small airway disease (SAD) has been increasingly studied in other interstitial lung diseases. For example, recent studies have indicated SAD as a possible negative prognostic factor in IPF, associated with accelerated functional decline and increased mortality [36]. Hypersensitivity pneumonia (HP) is also associated with small airway involvement, as evidenced by peribronchiolar inflammation due to bronchiolocentric infiltration by lymphocytes and granulomas. For this reason, SAD prevalence in HP patients was higher compared to the non-HP population, using impulse oscillometry (IOS) [37]. Cryptogenic Organizing Pneumonia (COP) is also associated with SAD, as its pathogenesis is characterized by an aberrant reaction to unknown injury in the small airways, resulting in peribronchiolar fibrosis and leading to small airway obstruction [38].

Finally, sarcoidosis can also be associated with SAD, as demonstrated by IOS and MEF_25_% [38]. This functional impairment may be due to granulomatous inflammation involving peribronchiolar lymphatics leading to bronchiolar obstruction and consequent air trapping [39]. Regarding sex-related differences in small airway function, previous studies have reported that the male sex was a risk factor associated with IPF comorbid SAD [36]. In contrast, in sarcoidosis, women showed a higher risk of developing airflow limitation on spirometry versus men [40]. In HP and COP, however, no significant sex-based differences in small airway function have emerged from previous studies.

### 4.2. Diffusing Capacity of the Lungs for Carbon Monoxide

DLCO measures the diffusion capacity of gas (carbon monoxide) across the alveolar–capillary interface and is sensitive to structural and functional alterations. It represents the area responsible for the effective exchange of respiratory gases and can therefore be reduced in conditions such as alveolar destruction (emphysema, pulmonary fibrosis, LCH, and sarcoidosis), interstitial lung diseases, dentoalveolar diseases (hemorrhages, edema, and pneumonia), or pulmonary atelectasis. In our cohort, women with LCH showed significantly lower %DLCO and %DLCO/VA than men (81 ± 22% vs. 92 ± 24% and 84 ± 18% vs. 94 ± 18%, respectively). This finding suggests that diffusing lung capacity, in addition to being reduced for the ventilatory inhomogeneity with an altered ventilation/perfusion (V/Q) ratio due to air trapping and small airway dysfunction, is also decreased due to the structural damage of the alveolar–capillary membrane by lung cists, nodules, and fibrotic remodeling. The lower %DLCO/VA values in women with LCH further supports the hypothesis of early alveolar–capillary membrane dysfunction in women with LCH as a key pathophysiological mechanism of DLCO reduction in this cohort. Notably, in the female LCH cohort, %DLCO was the functional parameter with the highest accuracy in the identification of lung involvement in multivariate analysis, with moderate discriminatory power (AUC: 0.70). This finding once again supports the hypothesis that alveolar–capillary membrane alterations may precede overt spirometry or N2-MBW parameters in LCH women. Our findings suggest that DLCO may be a moderately valuable functional marker of lung involvement in adult LCH females. However, the AUC of 0.70 indicates limited discriminatory performance, and external validation in independent cohorts is important to validate its clinical utility.

Sex-related differences in DLCO have also been explored in other ILDs. For example, in the Swedish IPF registry, female former smokers exhibited a higher mean %DLCO compared to male former smokers. However, this difference was not present among non-smokers [17]. In sarcoidosis, conversely, women had lower %DLCO and %DLCO/VA than men. This difference may be due to both more severe air trapping and sex-specific immune-mediated endothelial dysfunction at the alveolar–capillary membrane in women with sarcoidosis [40]. In COP and HP, however, there are no data on sex-based differences in DLCO, given the rarity of these diseases.

### 4.3. Sex-Based Functional Phenotypes: Clinical Implications

Our results indicate the presence of a distinct functional phenotype in women with LCH. Notably, women reported significantly lower %DLCO and %DLCO/VA values, together with a higher RV/TLC ratio, indicative of increased air trapping with the earlier impairment of diffusing lung capacity. In addition, a trend toward lower MEF_25_% and FEF_25–75_% values in women may further sustain small airway involvement. All these data suggest that the female functional LCH phenotype may be characterized by early diffuse alveolar–capillary dysfunction associated with early small airway dysfunction with consequent air trapping, in the absence of apparent restrictive or obstructive spirometric patterns. Furthermore, these differences were evident despite the same prevalence of smoking habits or lung involvement between women and men. This implies a possible sex-specific pathophysiological trajectory, potentially linked to hormonal, immunological, or endothelial mechanisms. Previous studies have shown that the hormones progesterone and estrogen increase venular and capillary permeability in the uterus or gingiva, causing endothelial damage [41,42]. In addition, a previous study reported that women had a lower diffusion capacity-to-cardiac output ratio (DLCO/Q˙), pulmonary capillary volume (Vc), and membrane diffusion capacity (Dm) than men of the same height during exercise [43]. However, no more sex-related differences were observed after correction for lung size [43]. Although there were no statistically significant differences between men and women in terms of symptoms, women exhibited exertional dyspnea more frequently (56.5% vs. 34.5%). This finding could further support the hypothesis of the female functional phenotype with an associated clinical phenotype (greater subjective sensation of breathlessness). However, these findings require prospective validation with standardized questionnaires in order to identify a sex-based clinical–functional phenotype in LCH. In conclusion, this functional female phenotype in LCH may represent an early and more compromised stage of pulmonary involvement that needs to be identified early and managed correctly.

### 4.4. SEX-Based Functional Phenotypes: Management Implications

The phenotypic functional features of LCH women in our cohort, revealing early small airway impairment with consequent air trapping and early reduction in carbon monoxide diffusion capacity, provide specific implications for the management and follow-up strategies of these patients. From a diagnostic perspective, spirometry alone may be measured at the initial evaluation, as patients can exhibit normal spirometric values with an isolated reduction in DLCO or an isolated increase in RV/TLC. A complete functional evaluation including DLCO and N2-MBW should therefore be performed. Early detection of these initial alterations may change the management and prognosis of this population, especially in LCH women.

From a management perspective, the female functional phenotype suggests the timely performance of accurate imaging such as HRCT to identify early signs of parenchymal lung involvement, especially in the presence of subtle respiratory symptoms such as exertional dyspnea. HRCT could serve as the baseline for future follow-up and patient’s severity stratification.

From a therapeutic perspective, although no specific recommendations for inhaled therapy exist in LCH with lung involvement, the female functional phenotype may benefit from the early use of inhaled bronchodilator therapy. In fact, the use of long-acting β2-agonists (LABAs) and/or inhaled corticosteroids (ICSs) could have a positive impact on the clinical and functional conditions of these patients, especially those with early air trapping or small airway dysfunction. This use has been proposed previously, but no study in the literature provides data on the effect of inhaled bronchodilator therapy in LCH patients with lung involvement [44].

From a follow-up perspective, the detection of early functional impairments in the female phenotype at the first evaluation could suggest a more careful follow-up strategy, with complete lung functional monitoring every 6 months for the first two years after diagnosis. This strategy could prevent or at least detect any functional deterioration at an early stage. However, prospective studies are needed to assess the impact of close follow-up on the decline in lung function in LCH patients with lung involvement. Finally, a multidisciplinary team comprising pulmonologists, radiologists, hematologists, and rehabilitation specialists may create the core for personalized medicine characterized by targeted and selected therapeutic interventions in LCH patients, especially women. In conclusion, our data on women with LCH suggest that personalized and careful management may have an impact on the functional state and quality of life of this population.

### 4.5. Study Strengths and Limitations

The present study is one of the largest monocentric studies focusing on adult LCH and, to our knowledge, is the first study to systematically investigate functional pulmonary phenotypes according to sex in LCH. Furthermore, the availability of comprehensive functional data by spirometry, N2-MBW, and DLCO in 79 consecutive adult real-life LCH patients is another notable point of this study. Another strength is the balanced number of women and men in our study, which allows us to perform statistical tests without losing analytical power. Furthermore, by including both LCH patients with and without lung involvement, we were able to identify the possible functional predictors of lung radiological involvement in LCH.

In terms of limitations, first of all, the present is a retrospective study, so our results may not be generally applicable in clinical practice. Furthermore, the lack of long-term follow-up data is a major limitation, as we cannot evaluate individual functional trajectories over time, especially for DLCO. Consequently, it is not possible to establish the prognostic value of the functional differences observed. For these reasons, our proposed female functional phenotype should be interpreted with caution and may be validated prospectively in independent cohorts.

The lack of specific clinical questionnaires such as the St. George’s Respiratory Questionnaire (SGRQ), modified Medical Research Council (mMRC) for dyspnea, and COPD Assessment Test (CAT) also limits us from identifying the clinical context of these patients and correlating the clinical picture with the functional one. Finally, a key limitation of this study is the lack of quantitative analysis at chest HRCT, such as cyst burden or nodularity scores. This lack limits us from correlating the radiological presentation with the functional one in LCH patients with lung involvement.

## 5. Conclusions

The present study provides the first comprehensive analysis of sex-based differences in lung function among adult patients with LCH. Our findings reveal the existence of a distinct female functional phenotype, characterized by lower DLCO (even when adjusted for VA) and a higher RV/TLC ratio despite a similar prevalence of lung involvement. In addition, a trend towards lower small airway spirometric parameters (such as MEF_25_% and FEF_25–75_%) supports the hypothesis that adult female LCH patients may exhibit earlier small airway impairment, resulting in air trapping, compared to males. These differences are unlikely to be explained only by age or smoking status, suggesting a possible sex-based pathophysiological mechanism, where adult women with LCH may suffer from earlier microvascular damage at the alveolar–capillary membrane compared to men, in addition to greater alveolar destruction due to air trapping (higher RV/TLC). Future studies investigating the role of hormonal, immunological, or genetic factors that may explain sex-based differences in the alveolar–capillary membrane are needed.

These results highlight the need for a sex-mediated approach for the pulmonary assessment in LCH patients. In LCH women, a complete functional evaluation including DLCO and N2-MBW should be considered, even in the case of normal spirometry, due to the higher incidence of women with isolated DLCO reduction or an increase in the RV/TLC ratio at the first functional evaluation in our cohort.

Our findings also support the idea of sex-based follow-up strategies, suggesting that LCH women with this female functional phenotype at diagnosis may require closer follow-up, in addition to targeted radiological examinations at regular intervals.

Although evidence on the therapeutic agents of lung function in LCH is very limited, our findings may suggest that women with early functional impairment may benefit from early bronchodilator therapy.

Future prospective studies are needed to validate our observations, particularly regarding the existence of the female functional phenotype in LCH, the underlying pathophysiological mechanisms, and the functional trajectories over time, also prospectively evaluating the impact of sex-based therapeutic and management strategies.

## Figures and Tables

**Table 1:** Cohort’s demographic and functional characteristics.

	Women (n = 47)	Men (n = 32)	*p*-Value
Age, years	45 ± 12	38 ± 12	0.007
Smoking habit, n (%)	smokers: 13 (28%)	smokers: 12 (37%)	NS
	former smokers: 25 (53%)	former smokers: 14 (44%)	
	no smokers: 9 (19%)	no smokers: 6 (19%)	
Lung involvement, n (%)	34 (72%)	20 (62%)	0.7
Pack years	12 ± 14	22 ± 21	0.021
Resting dyspnea, n (%)	12 (25.5%)	6 (19%)	NS
Exertional dyspnea, n (%)	26 (56.5%)	11 (34.5%)	NS
Cough, n (%)	18 (38%)	8 (25%)	NS
FEV_1_, L	2.7 ± 0.6	4.1 ± 1.3	<0.001
%FEV1	100 ± 20	100 ± 27	NS
FVC, L	3.4 ± 0.6	5.2 ± 1.2	<0.001
%FVC	109 ± 16	105 ± 21	NS
FEV_1_/FVC	0.8 ± 0.1	0.8 ± 0.1	NS
MEF_25_%	0.59 ± 0.31	0.7 ± 0.4	0.075
FEF_25–75_%	0.75 ± 0.3	0.88 ± 0.35	0.08
TLC, L	5.1 ± 0.9	6.9 ± 1.2	<0.001
%TLC	111 ± 19	109 ± 23	NS
RV, L	1.6 ± 0.6	2.6 ± 1	<0.001
%RV	102 ± 36	115 ± 40	NS
RV/TLC	0.35 ± 0.09	0.3 ± 0.08	0.005
FRC, L	2.3 ± 0.6	3.4 ± 1	<0.001
%FRC	98 ± 26	111 ± 34	NS
%DLCO	81 ± 22	92 ± 24	0.02
%DLCO/VA	84 ± 18	94 ± 18	0.01

Table 1: Values are reported as mean ± standard deviation. FEV_1_, forced expiratory volume in the first second; FVC, forced vital capacity; MEF_25_%, maximal expiratory flow at 25%; FEF_25–75_%, forced expiratory flow at 25–75%; TLC, total lung capacity; RV, residual volume; FRC, functional residual capacity; DLCO, diffusing capacity of the lungs for carbon monoxide; VA, alveolar volume; NS, not significant.

**Table 2:** Multivariable linear regression analysis for lung involvement (dependent variable) in LCH women.

Independent Variable	β Coefficient	OR (95% CI)	*p*-Value
Intercept	5.3	-	
Resting dyspnea	31.5	4.25 × 10^13^ (3.83 × 10^−6^–1.76 × 10^34^)	NS
Exertional dyspnea	1.3	3.39 × 10^0^ (3.98 × 10^−1^–2.89 × 10^1^)	NS
%FEV_1_	−42	3.75 × 10^−19^ (9.57 × 10^−54^–1.47 × 10^16^)	NS
%FVC	16	9.41 × 10^8^ (3.03 × 10^−12^–2.92 × 10^25^)	NS
RV/TLC	1.47	1.35 × 10^6^ (4.65 × 10^−5^–3.91 × 10^16^)	NS
%DLCO	−18	1.60 × 10^−8^ (2.09 × 10^−15^–1.22 × 10^−1^)	0.02
%DLCO/VA	−4	5.91 × 10^4^ (3 × 10^−10^–1.17 × 10^19^)	NS

LCH, Langerhans Cell Histiocytosis; OR, odds ratio; FEV_1_, forced expiratory volume in the first second; FVC, forced vital capacity; TLC, total lung capacity; RV, residual volume; FRC, functional residual capacity; DLCO, diffusing capacity of the lungs for carbon monoxide; VA, alveolar volume; NS, not significant.

**Table 3:** Multivariable linear regression analysis for lung involvement (dependent variable) in LCH men.

Independent Variable	β Coefficient	OR (95% CI)	*p*-Value
Intercept	17.17	-	
Resting dyspnea	2.55	1.27 × 10^12^ (1.30 × 10^−2^–1.90 × 10^6^)	NS
Exertional dyspnea	28	1.28 × 10^1^ (5.74 × 10^−1^–5.53 × 10^1^)	NS
%FEV_1_	−69	6.78 × 10^−31^ (4.42 × 10^−62^–1.04 × 10^1^)	0.06
%FVC	45.3	4.89 × 10^19^ (9.59 × 10^−6^–2.49 × 10^44^)	NS
RV/TLC	34.4	8.87 × 10^14^ (3.60 × 10^−9^–2.19 × 10^38^)	NS
%DLCO	−4.4	0.012 (2.71 × 10^−11^–5.50 × 10^8^)	NS
%DLCO/VA	−6.7	1.88 × 10^−6^ (5.54 × 10^−15^–636.36)	NS

LCH, Langerhans Cell Histiocytosis; OR, odds ratio; FEV_1_, forced expiratory volume in the first second; FVC, forced vital capacity; TLC, total lung capacity; RV, residual volume; FRC, functional residual capacity; DLCO, diffusing capacity of the lungs for carbon monoxide; VA, alveolar volume; NS, not significant.

## Data Availability

The dataset used for our analysis is available upon request to the corresponding author of this study.
